# Peroxisomes and peroxisomal transketolase and transaldolase enzymes are essential for xylose alcoholic fermentation by the methylotrophic thermotolerant yeast, *Ogataea (Hansenula) polymorpha*

**DOI:** 10.1186/s13068-018-1203-z

**Published:** 2018-07-19

**Authors:** Olena O. Kurylenko, Justyna Ruchala, Roksolana V. Vasylyshyn, Oleh V. Stasyk, Olena V. Dmytruk, Kostyantyn V. Dmytruk, Andriy A. Sibirny

**Affiliations:** 1 Department of Molecular Genetics and Biotechnology, Institute of Cell Biology, Drahomanov Str., 14/16, Lviv, 79005 Ukraine; 2 Department of Biotechnology and Microbiology, University of Rzeszow, Zelwerowicza 4, 35-601 Rzeszow, Poland

**Keywords:** Xylose, *Ogataea (Hansenula) polymorpha*, Peroxisomes, Ethanol, High-temperature alcoholic fermentation

## Abstract

**Background:**

*Ogataea (Hansenula) polymorpha* is one of the most thermotolerant xylose-fermenting yeast species reported to date. Several metabolic engineering approaches have been successfully demonstrated to improve high-temperature alcoholic fermentation by *O. polymorpha*. Further improvement of ethanol production from xylose in *O. polymorpha* depends on the identification of bottlenecks in the xylose conversion pathway to ethanol.

**Results:**

Involvement of peroxisomal enzymes in xylose metabolism has not been described to date. Here, we found that peroxisomal transketolase (known also as dihydroxyacetone synthase) and peroxisomal transaldolase (enzyme with unknown function) in the thermotolerant methylotrophic yeast, *Ogataea (Hansenula) polymorpha*, are required for xylose alcoholic fermentation, but not for growth on this pentose sugar. Mutants with knockout of *DAS1* and *TAL2* coding for peroxisomal transketolase and peroxisomal transaldolase, respectively, normally grow on xylose. However, these mutants were found to be unable to support ethanol production. The *O. polymorpha* mutant with the *TAL1* knockout (coding for cytosolic transaldolase) normally grew on glucose and did not grow on xylose; this defect was rescued by overexpression of *TAL2*. The conditional mutant, *pYNR1*-*TKL1*, that expresses the cytosolic transketolase gene under control of the ammonium repressible nitrate reductase promoter did not grow on xylose and grew poorly on glucose media supplemented with ammonium. Overexpression of *DAS1* only partially restored the defects displayed by the *pYNR1*-*TKL1* mutant. The mutants defective in peroxisome biogenesis, *pex3Δ* and *pex6Δ*, showed normal growth on xylose, but were unable to ferment this sugar. Moreover, the *pex3Δ* mutant of the non-methylotrophic yeast, *Scheffersomyces (Pichia) stipitis*, normally grows on and ferments xylose. Separate overexpression or co-overexpression of *DAS1* and *TAL2* in the wild-type strain increased ethanol synthesis from xylose 2 to 4 times with no effect on the alcoholic fermentation of glucose. Overexpression of *TKL1* and *TAL1* also elevated ethanol production from xylose. Finally, co-overexpression of *DAS1* and *TAL2* in the best previously isolated *O. polymorpha* xylose to ethanol producer led to increase in ethanol accumulation up to 16.5 g/L at 45 °C; or 30–40 times more ethanol than is produced by the wild-type strain.

**Conclusions:**

Our results indicate the importance of the peroxisomal enzymes, transketolase (dihydroxyacetone synthase, Das1), and transaldolase (Tal2), in the xylose alcoholic fermentation of *O. polymorpha*.

**Electronic supplementary material:**

The online version of this article (10.1186/s13068-018-1203-z) contains supplementary material, which is available to authorized users.

## Background

Peroxisomes are defined as organelles that typically contain the H_2_O_2_-generating enzymes, oxidase, and catalase. Most peroxisomes from yeasts to humans contain enzymes conducting fatty acid β-oxidation. Peroxisomes in fungi and plants also harbor some enzymes from the glyoxylic acid cycle [1–3]. However, the peroxisome is a metabolically versatile organelle containing enzymes catalyzing numerous catabolic and some biosynthetic reactions. In filamentous fungi, peroxisomes contain some enzymes conferring biotin and penicillin biosynthesis [4, 5]. In yeasts, peroxisomes are involved in the catabolism of many carbon and nitrogen sources, such as methanol, *n*-alkanes, purines, d-amino acids, methylamine, ethylamine, pipecolic acid, sarcosine, glycolate and spermidine [3, 6]. Involvement of peroxisomes in sugar catabolism has been described for some parasitic *Kinetoplastida* protists, where these organelles, also known as glycosomes, contain enzymes catalyzing key glycolytic reactions [7]. Regarding yeast and filamentous fungi, until recently it has been accepted that the enzymes of glucose catabolism (i.e., glycolysis and pentose phosphate pathway) are located in the cytosol [8]. However, recent studies on *Cryptococcus neoformans* and *Ustilago maydis* showed that some of the glycolytic enzymes exhibit dual localization in both cytosol and peroxisomes [9, 10]. Importantly, defects in peroxisomal localization of glycolytic proteins or deficient peroxisome biogenesis impaired growth of these organisms on glucose. *Candida albicans* and related species showed cytosolic and peroxisomal localization of enzymes from the oxidative branch of the pentose phosphate pathway, namely glucose-6-phosphate dehydrogenase and 6-phosphogluconate dehydrogenase [11]. Both cytosolic and peroxisomal isoforms of these proteins are encoded by single genes, suggesting that dual localization of the enzymes results from alternative splicing or ribosomal read-through of the stop codons [8, 10]. However, the methylotrophic yeast, *Komagataella phaffii (Pichia pastoris)*, contains two sets of enzymes from the non-oxidative segment of the pentose phosphate pathway. These are the cytosolic and peroxisomal isoforms, encoded by different paralogs [12]. It is also known that peroxisomal enzymes are involved in the so-called xylulose monophosphate pathway for formaldehyde assimilation during growth on methanol. It has been suggested that this pathway for formaldehyde assimilation relies solely on the peroxisomal (and not cytosolic) enzymes of the non-oxidative branch of the pentose phosphate pathway (e.g., transketolase, transaldolase, ribulose phosphate epimerase, and ribose phosphate isomerase). Putative functions of these peroxisomal isoforms in the xylose metabolism of *K. phaffii* were not studied.

Here, we addressed the metabolism and ethanol fermentation of the second most abundant sugar in nature, xylose. Xylose is the major component of the hemicellulose found in grasses and hard wood trees. For this study, we used the methylotrophic yeast, *Ogataea (Hansenula) polymorpha*, which is capable of fermenting glucose, cellobiose, and xylose [13]; as well as converting glycerol to ethanol [14]. Moreover, this organism is the most thermotolerant yeast known to date with a maximal growth and fermenting temperature of 50 °C, making it suitable for the process of simultaneous saccharification and fermentation [15]. Recombinant *O. polymorpha* strains expressing amylolytic and xylanolytic enzymes grow on starch and xylan, respectively. They also ferment these polymers to ethanol [16]. Unfortunately, the efficiency of xylose alcoholic fermentation by wild-type *O. polymorpha* strains is very low [17]. However, the efficiency of xylose to ethanol fermentation was significantly improved by a combination of classical selection and novel approaches in metabolic engineering. Interestingly, although it is possible to efficiently express bacterial xylose isomerase in *O. polymorpha* and the recombinant enzyme shows high specific activity, the ethanol yield remained low [17, 18]. Instead, an increase in ethanol production from xylose was achieved upon overexpression of the engineered xylose reductase [19] or pyruvate decarboxylase [20]. In addition, *O. polymorpha* strains with substantially improved ethanol production from xylose were obtained by overexpression of the modified *XYL1* gene and native *XYL2* and *XYL3* genes coding for enzymes used by the initial reactions of xylose metabolism [21]. Further increase in ethanol production from xylose was achieved by deletion of the *CAT8* gene coding for a putative transcription factor [22]. The best available strains accumulated nearly 12.5 g ethanol/L in xylose medium at 45 °C, which is 20 times greater than the wild-type strain, and yet inferior to recombinant strains of *Saccharomyces cerevisiae* and *Scheffersomyces (Pichia) stipitis* (which are mesophilic microorganisms). Further improvement of the high-temperature ethanol fermentation could be achieved by identification of the bottlenecks likely to exist in the xylose conversion to ethanol in the thermotolerant yeast, *O. polymorpha*.

**Fig. 1:**
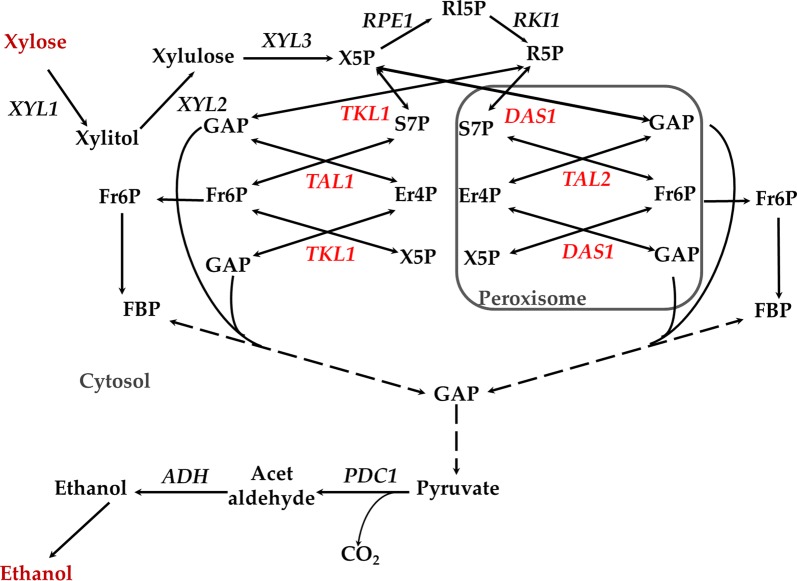
The metabolic pathway of xylose in *O. polymorpha.* X5P, xylulose 5-phosphate; R5, ribose 5-phosphate; RI5P, ribulose 5-phosphate; GAP, glyceraldehyde 3-phosphate; S7P, sedoheptulose 7-phosphate; Fr6P, fructose 6-phosphate; Er4P, erythrose 4-phosphate; FBP, fructose 1,6-bisphosphate; Genes: *XYL1*, xylose reductase; *XYL2*, xylitol dehydrogenase; *XYL3*, xylulokinase; *RPE1*, ribulose 5-phosphate epimerase; *RKI1*, ribose 5-phosphate isomerase; *TKL1*, transketolase; *TAL1*, translaldolase; *DAS1*, dihydroxyacetone synthase (peroxisomal transketolase); *TAL2*, peroxisomal transaldolase; *PDC1*, pyruvate decarboxylase; *ADH1*, alcohol dehydrogenase

Xylose metabolism in yeast consists of three parts: conversion to xylulose-5-phosphate, reactions of pentose phosphate pathway, and the glycolysis reactions (Fig. 1) [23]. In this case, the presence of all three parts is strongly confirmed by both biochemical and genetic evidence. It is also assumed that all corresponding enzymes are in the cytosol. However, it has been known for many years that methylotrophic yeast contains peroxisomal transketolase, known also as dihydroxyacetone synthase [24]. This enzyme is involved in the assimilation of formaldehyde during methylotrophic growth and also catalyzes the classical transketolase reaction (Fig. 1) [25]. Most of the peroxisomal proteins are located in peroxisomes due to the Peroxisome Targeting Signal I (PTS1), which represents the evolutionarily conserved amino acid sequence S–K–L [3, 26]. Due to the PTS1 sequence, dihydroxyacetone synthase from *O. polymorpha* is also located in the peroxisomes of the heterologous system for the non-methylotrophic yeast, *Saccharomyces cerevisiae* [27]. The newly available complete sequence of *O. polymorpha* genome (http://genome.jgipsf.org/Hanpo2/Hanpo2.home.html; [28]) has allowed identification of genes presumably encoding peroxisomal transaldolase [29]; as well as ribulose phosphate epimerase (K. Dmytruk, A. Sibirny, unpublished). Peroxisomal transaldolase, designated here as Tal2, contains a classical PTS1 signal S–K–L. Peroxisomal localization of *O. polymorpha* Tal2 was not directly determined before; however, its ortholog (Tal1-2) was found to be localized in peroxisomes in a closely related methylotrophic species, *K. phaffii* [12]. Homology in protein sequences between Tal2 *O. polymorpha* and Tal1-2 *K. phaffii* is rather high (63% identities, 79% positives) and the Tal2 protein of *O. polymorpha* also contains PTS1 signal. These data suggest that Tal2 of *O. polymorpha* is most likely to be a peroxisomal protein. However, direct evidence for peroxisomal localization of Tal2 was absent before this work.

Methylotrophic yeasts possess well-developed peroxisomes which could occupy up to 80% of cellular volume during growth on methanol [30]. We hypothesized that peroxisomal enzymes of the pentose phosphate pathway could be important not only for methanol metabolism [12], but also for xylose to ethanol fermentation (Fig. 1). We also decided to test the effect of *pex* mutations, which block peroxisome biogenesis on xylose growth and ethanol fermentation. We found that the corresponding knockouts in *das1, tal2, pex3*, or *pex6,* severely damaged xylose ethanol fermentation but did not affect growth on this sugar. Moreover, defects in expression of *TKL1* (cytosolic transketolase) suppressed growth on both xylose and glucose, whereas knockout of *TAL1* (cytosolic transaldolase) impaired only xylose growth. However, xylose fermentation was only partially suppressed in conditional *TKL1* (*pYNR1*-*TKL1*) and knockout *TAL1* (*tal1Δ*) mutants. In line with these observations, overexpression of the genes coding for cytosolic and peroxisomal transketolase and transaldolase (*TKL1, TAL1, DAS1, TAL2*) had an opposite effect and activated xylose alcoholic fermentation. Finally, simultaneous overexpression of *DAS1* and *TAL2* in the background of the recently isolated “best ethanol producer from xylose” [22] activated xylose alcoholic fermentation and enabled the construction of further improved ethanol producers from xylose at 45 °C.

## Methods

### Strains, media, and culture conditions

*Ogataea polymorpha* cells were grown on YPD (10 g/L yeast extract, 10 g/L peptone, 20 g/L glucose) or mineral medium (6.7 g/L YNB without amino acids, 20 g/L of glucose or xylose, or 10 g/L of methanol) at 37 °C. For the NCYC495 *leu1*-*1* strain, leucine (40 mg/L) was added to the medium. For selection of yeast transformants on YPD 0.1 g/L of nourseothricin, 0.3-0.5 g/L of geneticin, 0.075-0.1 g/L of zeocin, or 0.35 g/L of hygromycin were added. Ethanol fermentation of *O. polymorpha* strains was tested as described previously [22]. Fermentation experiments were performed in at least triplicate to ensure the results were reproducible. The bars in the figures indicate the ranges of the standard deviation.

Strains *pex3Δ* and *pex6Δ O. polymorpha* were kindly provided by Prof. Ida van der Klei, Groningen University, the Netherlands. *S. stipitis* strains—parental *ku80* [31] and *pex3*∆ mutants were grown on YNB medium supplemented with 40 mg/mL of histidine and 2 g/L glucose or 1 g/L of oleate at 30 °C. Ethanol fermentation of *S. stipitis* strains was tested (as mentioned above) for *O. polymorpha* strains under oxygen-limited conditions (100 rpm) at 30 °C.

The *E. coli* DH5α strain (Φ80d*lacZ*ΔM15, *recA*1, *endA*1, *gyrA*96, *thi*-1, *hsdR*17(r_K_^−^, m_K_^+^), *supE*44, *relA*1, *deoR*, Δ(*lacZYA*-*argF*)U169) was used as a host for plasmid propagation. Strain DH5α was grown at 37 °C in LB medium as described previously [32]. Transformed *E. coli* cells were maintained on a medium containing 100 mg/L of ampicillin.

### Molecular biology techniques

Standard cloning techniques were carried out as described [32]. Genomic DNA of *O. polymorpha* was isolated using the Wizard^®^ Genomic DNA Purification Kit (Promega, Madison, WI, USA). Restriction endonucleases and DNA ligase (Fermentas, Vilnius, Lithuania) were used according to the manufacturer specifications. Plasmid isolation from *E. coli* was performed with the Wizard^®^ *Plus* SV Minipreps DNA Purification System (Promega, Madison, WI, USA). DNA fragments were separated on a 0.8% agarose (Fisher Scientific, Fair Lawn, NJ, USA) gel. Isolation of fragments from the gel was carried out with a DNA Gel Extraction Kit (Millipore, Bedford, MA, USA). PCR amplification of the fragments of interest was done with Phusion^®^ High-Fidelity DNA Polymerase (Thermo Scientific, USA) according to the manufacturer’s specifications. PCRs were performed in GeneAmp^®^ PCR System 9700 thermocycler (Applied Biosystems, Foster City, CA, USA). Transformation of the yeasts *O. polymorpha* and *S. stipitis* was carried out as described previously [33].

### Construction and analysis of *das1Δ*, *tal2Δ*, *tal1Δ*, *pYNR1*-*TKL1 O. polymorpha* mutants

Genomic DNA of *O. polymorpha* NCYC495 *leu1*-*1* strain was used as a template for PCR amplification of 5′ and 3′ non-coding regions of *DAS1* using primers OL207/OL208 and OL209/OL210 (Additional file 1). The resulting 5′ *DAS1* (926 bp) and 3′ *DAS1* (1257 bp) fragments were *Hin*dIII/*Eco*RI or *Eco*RI/*Xba*I digested and cloned into *Hin*dIII/*Xba*I-linearized vector pBluescript II KS(−). The resulting recombinant construct was named pLRDAS. Gene zeoR (1132 bp) conferring resistance to zeocin was amplified using vector pPICZB (Thermo Fisher Scientific) as a template and primers Ko64/Ko65. The obtained fragment was *Eco*RI digested and cloned into *Eco*RI-linearized plasmid pLRDAS. The constructed plasmid was designated as p∆DAS-Zeo.

The same approach was used for construction of the *TAL2* deletion cassette. The 5′ (779 bp) and 3′ (1440 bp) non-coding regions of *TAL2* were PCR amplified with primers OL211/OL212 and OL213/OL214. Amplified 5′ and 3′ non-coding regions were double digested with *Hin*dIII/*Eco*RI and *Eco*RI/*Bam*HI and cloned into pBluescript II KS(−) digested with *Hin*dIII/*Bam*HI. Selective marker gene conferring resistance to zeocin was amplified from pPICZB with Ko64/Ko65 and cloned between 5′ and 3′ non-coding regions of *TAL2* as *Eco*RI digested fragment. The resulting plasmid was named p∆TAL-Zeo.

The constructed plasmids were transformed into *O. polymorpha* NCYC495 *leu1*-*1* recipient strain using an standard electroporation method [33]. Transformants were selected on the solid YPD medium supplemented with 75 mg/L of zeocin after 4 days of incubation. The obtained transformants were examined by PCR using genomic DNA of recombinant strains as a template. Transformants with confirmed deletion of *DAS1* and *TAL2* were stabilized by altering cultivation in non-selective and selective media and once again examined by PCR. Fragments with predicted size were amplified using pairs of primers homologous to the sequence of selective marker and regions outside from the fragments used for recombination (Ko533/Ko534, Ko557/Ko559 for *DAS1* and Ko535/Ko534, Ko557/Ko558 for *TAL2*) (Additional file 2).

The deletion cassette for isolation of *tal1Δ* mutant on the background of the wild-type strain was constructed as follows. Genomic DNA of *O. polymorpha* NCYC495 *leu1*-*1* strain was used as template for isolation of 5′ and 3′ non-coding regions of *TAL1* gene by PCR amplifications using primers OK74/OK75 and OK76/OK77. The obtained DNA fragments were fused by overlap PCR using primers OK74/OK77. The resulting fragment (1.8 kb) was *Bam*HI/*Kpn*I digested and cloned into the appropriate sites of pUC57 vector. The *hphNT1* gene (1777 bp), conferring resistance to hygromycin, was amplified from plasmid pRS42H [34] as a template and primers Ko446 and Ko450. The resulting plasmid was designated as p∆TAL1-Hygr.

The constructed plasmid was transformed into *O. polymorpha* NCYC495 recipient strain using the electroporation method given above [33]. Transformants were selected on solid YPD medium supplemented with 0.35 g/L of hygromycin after 4 days of incubation. Obtained transformants were examined by PCR using genomic DNA of recombinant strains as a template. Transformants with confirmed deletion of *TAL1* were stabilized by altering cultivation in non-selective and selective media and once again examined by PCR. Fragments with predicted size were amplified using pairs of primers homologous to the sequence of selective marker and regions outside from the fragments used for recombination (OK80/Ko450, Ko446/OK81). Additionally, primers OK99/OK100 for *TAL1* ORF amplification were used (Additional file 3).

The conditional knockout mutant *tkl1∆* was constructed by replacement of the endogenous promoter of *TKL1* gene by regulated *YNR1* promoter of nitrate reductase, repressed by ammonium sulfate as nitrogen source. Genomic DNA from *O. polymorpha* NCYC495 *leu1*-*1* strain was used as a template for PCR amplification of 5′ non-coding regions; and part of the ORF of *TKL1* gene using primers OK170/OK171 and OK174/OK175, respectively. The promoter of the *YNR1* gene was amplified from genomic DNA from the *O. polymorpha* NCYC495 *leu1*-*1* strain using primers OK172/OK173. The obtained DNA fragments were fused by overlap PCR using primers OK170 and OK175. The resulting fragment (3.6 kb) was *Kpn*I/*Sph*I digested and cloned into the corresponding sites of the pUC57 vector. Selective marker gene conferring resistance to hygromycin was amplified from plasmid pRS42H with primers Ko446 and Ko450 and cloned in *Xba*I-linearized plasmid pUC57-YNR1p_TKL1. The resulting plasmid was named pUC57-YNR1p_TKL1_ hphNT1.

The constructed plasmid was transformed into *O. polymorpha* NCYC495 recipient strain using the same electroporation method [33]. Transformants were selected on the solid YPD medium supplemented with 0.35 g/L of hygromycin after 4 days of incubation.

The correct replacement of *TKL1* gene promoter was confirmed by PCR in obtained transformants using primers OK176/OK123 and OK172/OK102 (Additional file 4).

### Construction and analysis of *O. polymorpha* strains with overexpression of *DAS1, TAL2, TAL1*, and *TKL1* genes

The recombinant plasmids, pGLG61/DAS1 and pGLG61/TAL2, bearing the *O. polymorpha* genes *DAS1* and *TAL2*, respectively, were constructed on the basis of the plasmid, pGLG61 [35].

Genomic DNA from *O. polymorpha* was used as a template for PCR amplifications of the *DAS1* and *TAL2* genes. Native promoters from these genes were substituted with a strong constitutive promoter, *GAP1,* from glyceraldehyde-3-phosphate dehydrogenase (GAP). First, the *GAP1* promoter and ORF of the *DAS1* gene with terminator sequence were amplified from *O. polymorpha* genomic DNA using the primers, Ko277/Ko278 and Ko279/Ko280. Then two fragments were fused by overlap PCR using primers, Ko277 and Ko280. The resulting fragment (2.9 kb) was digested with *Bam*HI endonuclease and ligated with *Bam*HI-linearized plasmid, pGLG61. The resulting recombinant construct was named pGLG61/DAS1 (Additional file 5A). At the next step, the *GAP1* promoter and ORF of the *TAL2* gene with terminator sequence were amplified from *O. polymorpha* genomic DNA using the primers, Ko405/Ko411 and Ko412/Ko414, respectively. Then two fragments were fused by overlap PCR using the primers, Ko405 and Ko414. The resulting fragment (1.8 kb) was digested with *Bam*HI and *Bgl*II endonuclease and ligated with the *Bam*HI/*Bgl*II-linearized plasmid, pGLG61. The resulting recombinant plasmid was named pGLG61/TAL2 (Additional file 5A). These plasmids were introduced into the genome of the *O. polymorpha* NCYC495 *leu1*-*1* strain. Transformants were selected on solid YPD medium supplemented with 0.3–0.5 g/L of geneticin after 5 days of incubation. Selected transformants were stabilized by alternating cultivation in non-selective and selective media and examined by diagnostic PCR using the primers, Кo277/Ko278 or Кo279/Кo280, respectively.

For overexpression of the cytosolic transketolase and transaldolase recombinant plasmids, pTkZr and pTaZr, bearing the *O. polymorpha* genes (*TKL1* and *TAL1)*, respectively, were constructed. The strong constitutive GAP promoter and ORF of the *TKL1* with terminator sequence were amplified by PCR from *O. polymorpha* chromosomal DNA using two pairs of primers, A58/A35 and Ko52/Ko53, respectively. The obtained PCR products were digested with *Sac*I/*Not*I and then ligated with *Sac*I/*Not*I-linearized vector pUC57, carrying the zeocin resistance gene as a selective marker (pUC57-Zr). The resulting plasmid was designated as pTkZr. For overexpression of the *TAL1* gene, the DNA fragment bearing the *GAP1* promoter was fused with the ORF of the *TAL1* gene. This was accomplished by overlap PCR using two pairs of primers, K43/Ko77 and Ko76/Ko84. The PCR product was digested with *BamH*I and then ligated with *Bam*HI-linearized vector, pUC57-Zr, resulting in the recombinant construct, pTaZr (Additional file 5B). The resulting plasmids were used for transformation of *O. polymorpha* NCYC495 *leu1*-*1* strain. The transformants were selected on solid YPD medium supplemented with 0.1 g/L of zeocin after 3 days of incubation.

For simultaneous overexpression of the *DAS1* and *TAL2* genes, the plasmid, pUC57/DAS1/TAL2, was constructed. *Bam*HI/*Pst*I-restriction fragment containing *GAPpr*-*TAL2* was isolated from the plasmid, pGLG61/TAL2, and cloned into the *Bam*HI/*Pst*I-linearized vector pUC57. The resulting recombinant construct was named pUC57/TAL2. The *Bam*HI-restriction fragment containing *GAPpr*-*DAS1* was isolated from the plasmid, pGLG61/DAS1, and cloned into the *Bam*HI-linearized and dephosphorylated vector, pUC57/TAL2. The resulting recombinant construct was named pUC57/DAS1/TAL2. Gene *natNT2*, conferring resistance to nourseothricin, was amplified from the plasmid, pRS41N [33] by PCR using primers, OK42/OK43. The fragment obtained (1.3 kb) was *Nde*I-linearized and cloned into the appropriate site of pUC57/DAS1/TAL2. The resulting plasmid was named pUC57/DAS1/TAL2/NTC (Additional file 5C). Transformants were selected on solid YPD medium supplemented with 0.1 g/L of nourseothricin after 3 days of incubation. Selected transformants were stabilized by alternating cultivation in non-selective and selective media and examined by diagnostic PCR using the primers, Ko277/Ko278 and Ko279/Ko280, respectively.

### Construction and analysis of *pex3Δ S. stipitis* mutants

Genomic DNA of *S. stipitis ku80* strain was used as template PCR amplification of 5′ and 3′ non-coding regions of *PEX3* gene using the primers, Ko770/Ko771 and Ko772/Ko773. The resulting 5′*PEX3* (1682 bp) fragment was *Sac*I/*Xba*I digested and cloned into *Sac*I/*Xba*I-linearized vector pUC57. The resulting plasmid was named pUC57-5′PEX3. As the next step, *3′PEX3* (1499 bp) fragments were *Xba*I/*Bam*HI digested and cloned into *Xba*I/*Bam*HI-linearized vector pUC57-5′PEX3. The resulting plasmid was named pUC57-5′-3′PEX3. *HIS3* gene (1399 bp) involved in histidine biosynthesis was amplified using vector p∆atg13Sc [36] as a template and primers KB5 and KB6. The obtained fragment was *Xba*I digested and subcloned into *Xba*I-linearized plasmid pUC57-5′-3′PEX3. As a result, the recombinant plasmid pUC57-5′-3′PEX3-HIS3 was constructed. This plasmid was *Pst*I-linearized and transformed into *S. stipitis ku80 his3*-*1* recipient strain using electroporation method [33]. Transformants were selected on solid YNB medium supplemented with 40 mg/L of histidine after 5 days of incubation at 30 °C. The obtained transformants were examined by PCR using genomic DNA of recombinant strains as a template. Transformants with confirmed deletion of *PEX3* were stabilized by altering cultivation in non-selective and selective media and once again examined by PCR. Fragments with predicted size were amplified using pairs of primers homologous to the sequence of selective marker and regions outside from the fragments used for recombination (Ko774/KB9 and KB10/Ko775) (Additional file 6).

### Fluorescence microscopy visualization of peroxisomal localization of Tal2 in *O. polymorpha*

For constitutive expression of peroxisomal SKL-tagged green fluorescent protein (GFP-SKL) in *O. polymorpha*, the DNA fragments harboring the GFP-SKL gene and the promoter *ScTEF1* were PCR amplified with the primers, Ko799/Ko800, from the plasmid, p416TEF-GFP [37]. The terminator, *ScCYC1,* was amplified with the primers, Ko801/Ko802, from the genomic DNA of *S. cerevisiae* BY4742. The backbone plasmid containing *KamMX4* selective marker was amplified with primers, Ko803/Ko804, from the plasmid, pCfB2055 [38]. Three PCR fragments were then Gibson assembled to generate the plasmid, pGFP-SLK. The obtained plasmid was digested with *Not*I and integrated into genome of *O. polymorpha* NCYC495 *leu1*-*1* strain. Transformants were selected on the solid YPD medium supplemented with 0.3 g/L of geneticin after 4 days of incubation. One of the transformants was used as a recipient strain for localization studies of Tal2 protein. The DNA fragment harboring the gene coding for the red fluorescent protein (RFP) was amplified with the primers, Ko888/Ko889, from the plasmid, pDsRed2 (Clontech). The backbone plasmid containing *TAL2* was amplified with the primers, Ko890/Ko891, from the plasmid, pGLG61/TAL2. Two PCR fragments were then Gibson assembled to generate the plasmid pGLG61/TAL2_RFP. This plasmid was introduced into genome of *O. polymorpha* wild-type strain carrying GFP-SKL. Transformants were selected on solid YNB medium after 5 days of incubation. Selected transformants were stabilized by alternating cultivation in non-selective and selective media and examined by diagnostic PCR using the primers, Ko892/Ko414. The resulting strains were grown at 37 °C in YNB medium with glucose, xylose, or methanol during 24 h; followed by microscopy analysis. Images were captured on a fluorescence microscope (Axio Imager A1; Carl Zeiss MicroImaging, Jena, Germany) coupled to a monochrome digital camera (Axio Cam MRm; Carl Zeiss MicroImaging) and processed using the AxioVision 4.5 (Carl Zeiss MicroImaging) and Adobe Photoshop CC software (Adobe Systems, Mountain View, CA).

### Biochemical methods

The enzyme activity was measured directly after the preparation of cell-free extracts. Protein concentration was determined with Folin reagent [39]. The specific activities of total transketolase and transaldolase in cell extracts were determined spectrophotometrically as described before [25, 40].

All assay experiments were repeated at least twice.

### Quantitative real-time PCR (qRT-PCR)

Expression of the *XYL1*, *XYL2*, *XYL3*, *RPE1*, *TKL1*, *PDC1*, *ADH1*, and *CYC1* genes was analyzed by real-time PCR. The qRT-PCR was performed by 7500 Fast Real-Time PCR System (The Applied Biosystems, USA) with SG OneStep qRT-PCR kit (EURx Ltd., Gdansk, Poland) using gene-specific pairs of primers, RNA as a template, and ROX reference passive dye according to the manufacturer’s instructions as described previously [22]. The primers pairs used for qRT-PCR are listed in Additional file 1. Sequences of the tested genes were taken from *O. polymorpha* genome database (*Ogataea polymorpha* NCYC 495 leu1.1 v2.0-JGI Genome Portal. http://genome.jgipsf.org/Hanpo2/Hanpo2.home.html).

### Analyses

The biomass was determined turbidimetrically with a Helios Gamma spectrophotometer (OD, 590 nm; cuvette, 10 mm) with gravimetric calibration. Concentrations of xylose and ethanol from fermentation in medium broth were analyzed by HPLC (PerkinElmer, Series 2000, USA) with an Aminex HPX-87H ion-exchange column (Bio-Rad, Hercules, USA). A mobile phase of 4 mM H_2_SO_4_ was used at a flow rate 0.6 mL/min and the column temperature was 30 °C. Alternatively, concentrations of ethanol in the medium were determined using alcohol oxidase/peroxidase-based enzymatic kit “Alcotest” [41]. Experiments were performed at least twice.

## Results

**Fig. 2:**
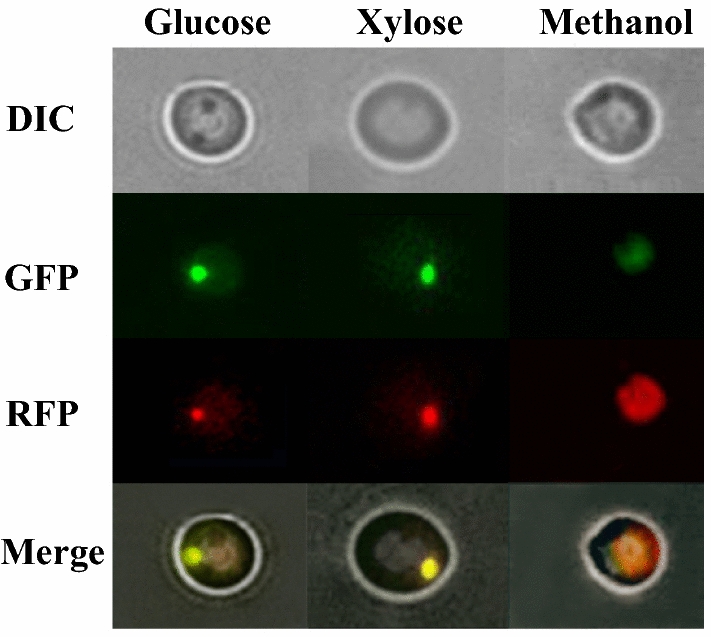
Fluorescent microscopic observation of peroxisomal localization analysis of Tal2. Fluorescence microscopy of *O. polymorpha* cells of the wild-type strain, which expresses GFP- PTS1 for labeling of peroxisomes and RFP for labeling of Tal2, grown in the liquid YNB medium with glucose (2%), xylose (2%), or methanol (1%) for 24 h

*Ogataea polymorpha* is a methylotrophic yeast and thus it contains a xylulose monophosphate pathway for formaldehyde assimilation, i.e., its conversion to three carbon compound dihydroxyacetone [42]. Work in *K. phaffii* showed that the key (if not all) enzymes of this pathway are located in peroxisomes [12, 29]. However, the functions of peroxisomal transketolase (dihydroxyacetone synthase), the key enzyme in the xylulose monophosphate pathway for xylose growth and fermentation have not been studied. As was pointed above, genome of *O. polymorpha* also contains the transaldolase paralog gene (designated here as *TAL2*), which contains codons for peroxisome targeting signal PTS1 (amino acids SKL). However, localization of this protein was not determined earlier. To study localization of the Tal2 protein, the *TAL2* gene was fused with the gene coding for RFP. The vector containing the *TAL2*-*RFP* fusion was introduced into the *O. polymorpha* wild-type strain, which expresses GFP containing the PTS1 signal. The GFP recipient strain we constructed possesses green fluorescent peroxisomes due to the targeting of GFP to this organelle, exactly as was described before [43] (not shown). Transformants of the GFP-expressed strain, which contained the *TAL2*-*RFP* fused gene, have been cultivated in the media with one of three carbon sources: glucose, xylose, and methanol. Cell fluorescence was also studied. Cells grown on each carbon source displayed both green and red fluorescence. Zones of green fluorescence corresponding to peroxisomes were the smallest in glucose-grown cells and the largest in methanol-grown cells, as expected. Most importantly, the red fluorescence of transaldolase Tal2 coincided perfectly with the green fluorescence of peroxisomes (Fig. 2). These data show that indeed transaldolase Tal2 containing PTS1 targeting signal is located only in peroxisomes, as expected.

**Fig. 3:**
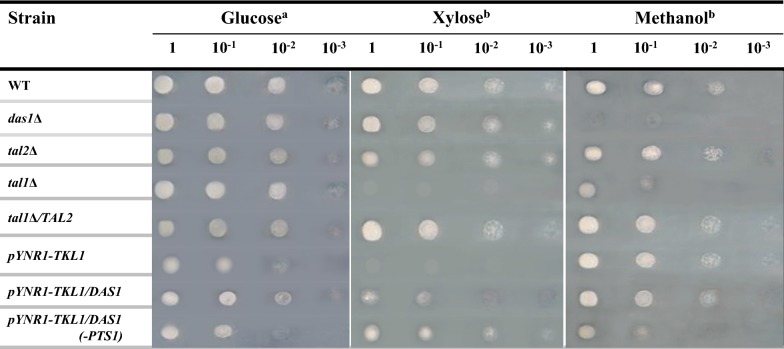
Growth of the *O. polymorpha* wild-type strain (WT) and strains *das1Δ*, *tal2Δ*, *tal1Δ*, *tal1Δ/TAL2*, *pYNR1*-*TKL1/DAS1*, *pYNR1*-*TKL1/DAS1(*-*PTS1)* on different carbon sources **a** on the second day of cultivation; **b** on the 5th day of cultivation; at 37 °C

**Fig. 4:**
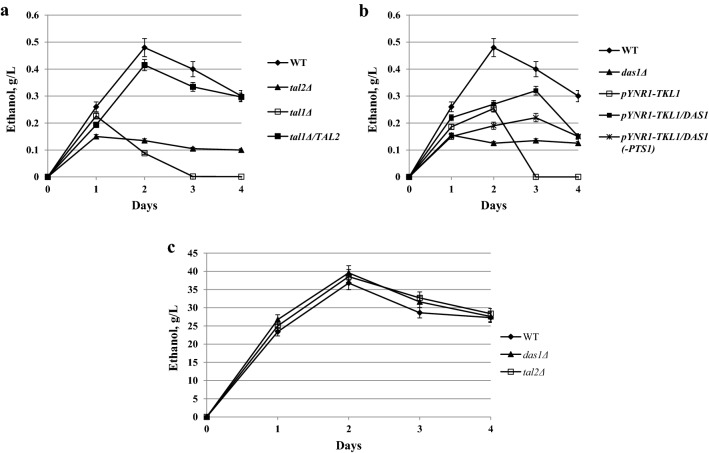
Ethanol production during xylose alcoholic fermentation of *O. polymorpha* strains **a** WT, *tal2Δ*, *tal1Δ*, *tal1Δ/TAL2*; **b** WT, *das1Δ*, *pYNR1*-*TKL1, pYNR1*-*TKL1/DAS1; pYNR1*-*TKL1/DAS1(*-*PTS1)*. Data are shown as mean of three independent experiments. **c** Ethanol production during glucose alcoholic fermentation of *O. polymorpha* strains WT, *das1Δ* and *tal2Δ*. Data are shown as mean of two independent experiments

The role of this peroxisomal transaldolase, Tal2; as well as the peroxisomal transketolase, Das1, in xylose metabolism and fermentation (and in this case of Tal2, also in methanol metabolism) was not studied. Furthermore, the roles of the cytosolic transketolase and transaldolase in xylose metabolism and fermentation in *O. polymorpha* have not been investigated as well. In this study, we have engineered knockout and overexpression strains for each corresponding gene (*DAS1, TAL2, TKL1, TAL1*) and studied their growth and fermentation on xylose and glucose. Mutants with knockout of genes coding for the peroxisomal transketolase, *DAS1,* and presumably the peroxisomal transaldolase, *TAL2,* did not differ from the wild-type strain regarding growth on glucose and xylose. Of note, mutant *das1Δ* failed to grow on methanol, whereas growth of *tal2Δ* mutant on methanol was normal (Fig. 3). However, the ability to ferment xylose was severely damaged in *tal2Δ* and *das1Δ* mutants (Fig. 4a, b). They also normally fermented glucose (Fig. 4c). The role of peroxisomal transketolase in methanol utilization has been known for many years [30]. Importantly, using *O. polymorpha tal2Δ* mutant, we did not observe the involvement of peroxisomal transaldolase in methanol metabolism (Fig. 3), unlike what was recently hypothesized for *K. pastoris* [12].

Subsequently, we decided to create deletion mutants in the genes coding the cytosolic transketolase and transaldolase enzymes. We found that the isolated *tal1Δ* mutant, in contrast to *tal2Δ* mutant, failed to grow on xylose, whereas growth on glucose was normal and growth on methanol was delayed (Fig. 3). Interestingly, despite complete inability to grow on xylose, *tal1Δ* mutant was still able to produce ethanol from this pentose (Fig. 4a). Next, we decided to test whether or not overexpression of the *TAL2* gene coding for peroxisomal transaldolase (under control of strong constitutive promoter, *GAP1,* for the gene coding for glyceraldehyde-3-phosphate dehydrogenase) could rescue *tal1Δ* growth on xylose. Indeed, transformants showed a restored ability to grow on xylose (Fig. 3). Moreover, xylose fermentation of the tested transformant was the same as in the wild-type strain (Fig. 4a).

**Fig. 5:**
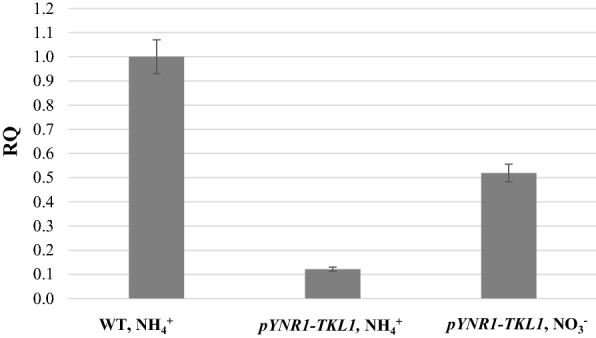
qRT-PCR assay of *TKL1* gene expression in WT and *pYNR1*-*TKL1* strains at the first day of xylose fermentation at 45 °C. RQ, relative quantity. Data are shown as mean of two independent experiments

Our multiple attempts to obtain mutants with knockout of the *TKL1* gene, encoding cytosolic transketolase were unsuccessful; even upon selection in the presence of aromatic and branched chain amino acids [44]. We speculated that *tkl1Δ* mutants of *O. polymorpha* could be lethal. Therefore, we decided to generate a conditional mutant expressing the *TKL1* gene under control of strongly inducible promoter and substituted the native *TKL1* promoter with that of *O. polymorpha YNR1* promoter of nitrate reductase gene, known to be repressed by ammonium and strongly induced by nitrate [45]. The obtained transformants grew (although poorly) on the mineral medium (with ammonium sulfate) and glucose as carbon source. The addition of aromatic and branched amino acids did not affect the growth of isolated transformants in the medium with glucose and ammonium sulfate (not shown). We then confirmed the inducible *TKL1* gene regulation under control of *YNR1* promoter by qRT-PCR. As expected, it was induced by nitrate and repressed by ammonium ions (Fig. 5).

However, in the medium with ammonium sulfate and xylose, transformants failed to grow at all (Fig. 3). Growth of the transformants on xylose and glucose media with nitrate as nitrogen source was normal (not shown). Thus, cytosolic transketolase is compulsory for xylose metabolism in *O. polymorpha*. It is interesting to note that growth of *pYNR1*-*TKL1* on methanol was unimpaired in contrast to that of the *tal1Δ* mutant, which showed growth defects on methanol (Fig. 3). Xylose fermentation of the *pYNR1*-*TKL1* strain was suppressed, but not fully abolished as in the case of *das1Δ* mutants (Fig. 4b).

We then tried to rescue *pYNR1*-*TKL1* strain’s growth on xylose by overexpression of *DAS1*. In this case, however, suppression was not complete as growth started after long lag-phase and was delayed (Fig. 3). We also constructed the shortened version of *DAS1* gene lacking C-terminal PTS1 motif. Overexpression of this presumably cytosolic Das1 protein more efficiently restored growth of *pYNR1*-*TKL1* mutant in the medium with xylose and ammonium sulfate (Fig. 3). Xylose fermentation of rescue strains *pYNR1*-*TKL1/DAS1* and *pYNR1*-*TKL1/DAS1(*-*PTS1)* is shown in Fig. 4. Like growth, ethanol production was also better rescued by the peroxisomal Das1 than its cytosolic counterpart. Still, ethanol production by both rescued transformants was approximately twofold lower on the second day of fermentation as compared to that of the wild-type strain (Fig. 4). Altogether, we conclude that peroxisomal transketolase and peroxisomal transaldolase are compulsory for xylose alcoholic fermentation, but not for growth on this pentose, whereas their cytosolic counterparts are required for xylose utilization.

**Fig. 6:**
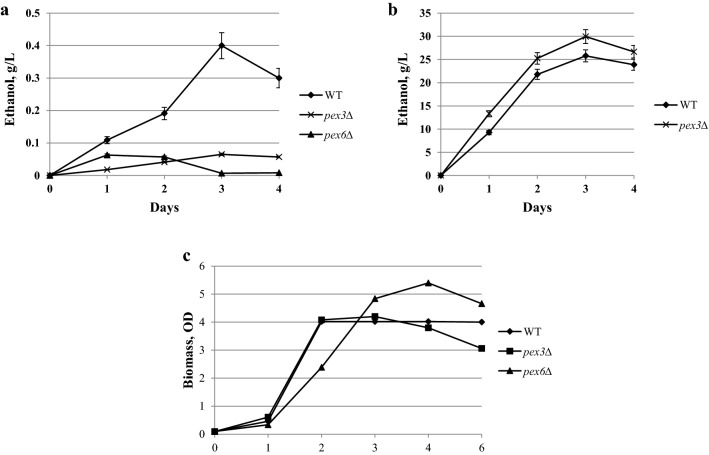
Ethanol production during **a** xylose or **b** glucose fermentation of *O. polymorpha* strains WT, *pex3Δ* and *pex6Δ*. Data are shown as mean of three independent experiments. **c** Growth of the *O. polymorpha* strains *pex3Δ* and *pex6Δ* on xylose as a carbon source as compared to the wild-type strain (WT). The data represent values of typical single cultivation

Next, we studied xylose growth and fermentation of the mutants defective in peroxisome biogenesis. We used mutants defective in the core membrane peroxisomal protein, Pex3, as well as the AAA-family ATPase, Pex6. The cells of *pex3Δ* and *pex6Δ* mutants *O. polymorpha* do not contain peroxisomes at all or contain only their remnants [3]. We found that growth of *pex3Δ* and *pex6Δ* mutants on xylose was normal, whereas xylose fermentation was totally abolished. However, ethanol production from glucose in *pex3Δ* mutants was close to that in the wild-type strain (Fig. 6). Thus, characteristics of xylose growth and fermentation of *O. polymorpha pex3Δ* and *pex6Δ* mutants were like those of *das1Δ* and *tal2Δ* mutants.

**Fig. 7:**
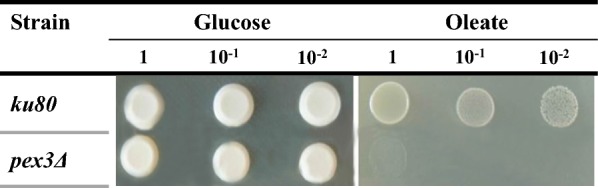
Growth of the *S. stipitis* mutant with deletion of *PEX3* gene on YNB medium with glucose (2%) or oleate (1%) relative to the wild-type strain *ku80* on the fifth day of cultivation at 30 °C

Inability of the mutants defective in peroxisome biogenesis to ferment xylose could be a consequence of mis-localization of peroxisomal transketolase and transaldolase into the cytosol. Alternatively, there could be an additional intrinsic requirement of peroxisome for xylose fermentation. To evaluate these possibilities, we decided to isolate peroxisome-deficient mutants in non-methylotrophic xylose-fermenting yeast, *S. stipitis*, whose genome does not contain genes of presumably peroxisomal transketolase and transaldolase. We created *S. stipitis* mutants with the *PEX3* knockout and they were unable to grow on oleic acid as a sole carbon source (Fig. 7).

**Fig. 8:**
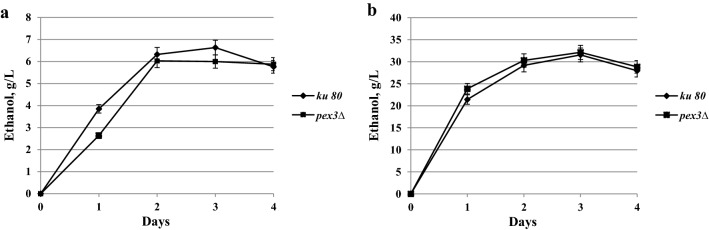
Ethanol production by parental *ku80* and deletion *pex3∆* strains of *S. stipitis*: **a** during xylose fermentation; **b** during glucose fermentation at 30 °C. Data are shown as mean of two independent experiments

Crucially, we found that unlike the *O. polymorpha pex3Δ*, the mutant of *S. stipitis* did not differ from the wild-type strain in terms of growth and fermentation of glucose and xylose (Fig. 8). Thus, in the non-methylotrophic yeast, *S. stipitis,* peroxisomes are not required for xylose fermentation, in contrast to the methylotrophic yeast, *O. polymorpha*. Possibly, this difference stems from the involvement of peroxisomal transketolase and transaldolase in regulation of xylose fermentation in *O. polymorpha*. Indeed, in *O. polymorpha pex3Δ* or *pex6Δ* mutants, these enzymes are mis-localized to the cytosol, where they presumably cannot fulfil their functions. On the other hand, in *S. stipitis,* an organism lacking peroxisomal transketolase and transaldolase, xylose fermentation does not require any peroxisomal enzymes.

Based on the data for the requirements of the peroxisomal transketolase (Das1) and the peroxisomal transaldolase (Tal2), we hypothesized that overexpression of *DAS1* and/or *TAL2* would have beneficial effects on ethanol production from xylose. To test this, we overexpressed each of the mentioned genes separately or together under control of the strong constitutive promoter, GAP. Overexpression of these genes led to an increase in the total activities of the corresponding enzymes, cytosolic and peroxisomal transketolase and transaldolase (Table 1).

**Table 1:** Specific activities of Tkl (transketolase) and Tal (transaldolase) in the cells of *O. polymorpha* strains with overexpressed *DAS1* and *TAL2* genes during cultivation at 37 °C

Activity	mU	
	Tkl	Tal
WT	10 ± 1.1	183 ± 2.33
WT/DAS1	21 ± 1.5	–
WT/TAL2	–	292 ± 3.45
WT/DAS1/TAL2	19 ± 0.5	288 ± 3.11

–, not determined

**Fig. 9:**
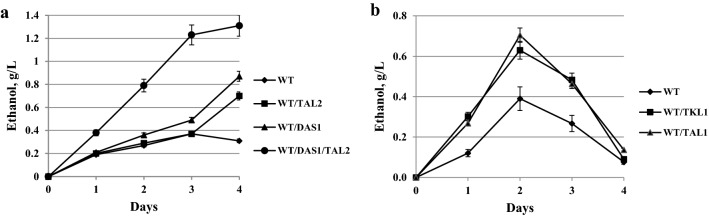
Ethanol production during xylose alcoholic fermentation at 45 °C by *O. polymorpha* strains **a** WT and strains with overexpression of *DAS1, TAL2,* co-overexpression of *DAS1* and *TAL2*; **b** WT and strains with overexpression of *TKL1, TAL1*. Data are shown as mean of three independent experiments

Transformants with overexpression of *TAL2* doubled their ethanol production from xylose, whereas overexpression of *DAS1* led to tripled ethanol production. Moreover, co-overexpression of *DAS1* and *TAL2* led to fourfold increase in ethanol production from xylose relative to that of the wild-type strain (Fig. 9a).

For comparison, we also overexpressed the genes, *TKL1* and *TAL1*, coding for cytosolic transketolase and transaldolase, respectively. We found that overexpression of these genes also stimulated ethanol production from xylose during 3 days of fermentation (Fig. 9b).

One may assume that Das1 and Tal2 proteins, in addition to their catalytic reactions, also affect the expression of other genes. We tested expression of several genes involved with xylose metabolism and ethanol fermentation (*XYL1, XYL2, XYL3, TKL1, TAL1, RPE1, CYC1, FBP1, PCK1, PDC1, ADH1*) in the strains with knockout and overexpression conferred by the *DAS1* and *TAL2* genes. Interestingly, this comparison showed that deletion of *DAS1* and *TAL2* genes lowered the expression of the *XYL1* gene coding for xylose reductase, whereas their overexpression, inversely, activated *XYL1* expression (Table 2). This result suggests possible involvement of peroxisomal transketolase and transaldolase in the transcriptional regulation of the xylose reductase (i.e., the enzyme of the initial step of xylose metabolism).

**Table 2:** Expression levels of the genes in strains with deletion or overexpression of *DAS1* or *TAL2* relative to the parental wild-type strain on the third day of xylose alcoholic fermentation at 45 °C

Strains	Genes									
	*XYL1*	*XYL2*	*XYL3*	*PDC1*	*RPE1*	*TKL1*	*ADH1*	*CYC1*	*FBP1*	*PCK1*
*das1∆/*WT	0.295 ± 0.072	1.330 ± 0.044	1.491 ± 0.111	1.253 ± 0.110	1.261 ± 0.267	1.384 ± 0.169	0.861 ± 0.032	1.397 ± 0.065	1.738 ± 0.056	1.462 ± 0.019
DAS1*/WT	2.155 ± 0.232	1.044 ± 0.134	0.801 ± 0.015	0.713 ± 0.006	0.877 ± 0.056	1.337 ± 0.041	0.571 ± 0.125	1.099 ± 0.088	0.309 ± 0.099	0.447 ± 0.098
*tal2∆*/WT	0.124 ± 0.062	1.538 ± 0.016	0.909 ± 0.181	0.717 ± 0.104	0.830 ± 0.072	0.867 ± 0.111	0.609 ± 0.128	1.576 ± 0.205	1.901 ± 0.049	1.44 ± 0.051
TAL2*/WT	1.452 ± 0.234	1.301 ± 0.246	1.158 ± 0.092	0.580 ± 0.101	0.575 ± 0.161	0.667 ± 0.122	1.246 ± 0.081	1.689 ± 0.133	1.428 ± 0.085	0.837 ± 0.114

The mRNA quantification was normalized to *ACT1* mRNA

We hypothesized that overexpression of *DAS1* and *TAL2* could be a useful approach for construction of advanced ethanol producers from xylose under elevated temperatures. To test this, we co-overexpressed the *DAS1* and *TAL2* genes in the background of the best previously constructed ethanol producer, BEP *cat8Δ* of *O. polymorpha* [22]. The resulting strain BEP *cat8*Δ/*DAS1/TAL2* accumulated 29% more ethanol relative to the parental strain BEP *cat8Δ* during xylose fermentation producing 16.1 g/L of ethanol versus 12.5 g/L for BEP *cat8Δ* [22]. The BEP *cat8*Δ/*DAS1/TAL2* strain accumulated only trace amounts of xylitol (up to 0.5 g/L) during xylose fermentation.

**Fig. 10:**
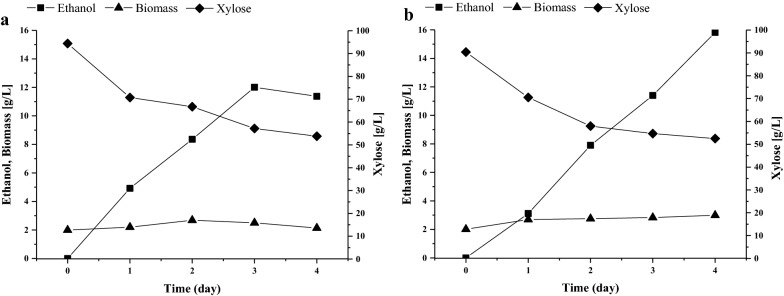
Ethanol production, biomass accumulation, and xylose consumption during xylose alcoholic fermentation at 45 °C by *O. polymorpha* strains **a** BEP *cat8*Δ and **b** BEP *cat8*Δ/*DAS1*/*TAL2*. The data represent values of typical single cultivation

These transformants did not demonstrate a change in ethanol production in glucose medium (not shown). Transformants with co-overexpression of *DAS1* and *TAL2* were characterized by increased ethanol production from xylose; however, xylose consumption from the medium was not activated relative to the parental strain (Fig. 10). The resulting strain exhibited a one-third increase in ethanol yield in xylose medium (Table 3) relative to the parental strain.

**Table 3:** Main parameters of xylose fermentation at 45 °C by the tested *O. polymorpha* strains

Strain	Ethanol (g/L)	Ethanol yield (g/g consumed xylose)	Rate of ethanol synthesis (g/g biomass/h)	Productivity of ethanol synthesis (g/L/h)
BEP *cat8*Δ	12.51 ± 0.134	0.340 ± 0.015	0.091 ± 0.003	0.205 ± 0.009
BEP *cat8*Δ/DAS1/TAL2	16.132 ± 0.141	0.393 ± 0.017	0.092 ± 0.004	0.210 ± 0.005

## Discussion

The role of peroxisomes in yeast and fungal glucose metabolism was recently established for relatively limited number of species [8–11]. However, nothing was known about the role of peroxisomes in the metabolism of the major pentose sugar, xylose. In this work, we have shown the importance of peroxisomal enzymes of PPP in xylose alcoholic fermentation in the native xylose-fermenting yeast, *O. polymorpha*. It was known for many years that this organism contains peroxisomal transketolase (dihydroxyacetone synthase, Das1) involved in methanol metabolism at the stage of formaldehyde assimilation [24]. Here, we found in the *O. polymorpha* genome database a gene designated (by us), as *TAL2,* which contains the PTS1 signal sequence and encodes the peroxisomal transaldolase (Tal2). This protein is located in peroxisomes under cultivation on glucose, xylose, or methanol as carbon sources (Fig. 2). It is interesting that both peroxisomal transketolase and transaldolase are not necessary for growth on xylose as a sole carbon and energy source, in contrast to their cytosolic counterparts, Tkl1 and Tal1, but are required for xylose to ethanol fermentation. Inversely, the knockout of genes coding for cytosolic transketolase, *TKL1,* and transaldolase, *TAL1,* totally abolished growth on xylose, while only partially suppressing the fermentation of this sugar. It should be stated that peroxisomal transaldolase is not involved in methanol metabolism in *O. polymorpha* (Fig. 3). Earlier, the role of peroxisomal transaldolase in methanol metabolism was suggested for *K. phaffii* [12]; however, such role was not shown directly.

We have also found that overexpression of Das1 and/or Tal2 enhanced ethanol production from xylose (like the effects of Tkl1 and Tal1 overexpression). Importantly, these manipulations specifically affected xylose fermentation, but not that of glucose. A crucial difference between growth and fermentation is that the latter demands limited oxygen supply. It could be that Das1 and Tal2 might be involved in responding to limited oxygen. Reactions catalyzed by transketolase and transaldolase do not involve any oxidoreductive changes; however, it is still possible that further peroxisomal metabolism decreases cell reductive state, which is favorable for xylose (but not glucose) alcoholic fermentation. Alternatively, this result could be explained by the potential involvement of the *DAS1* and *TAL2* genes in transcriptional regulation of *XYL1*, coding for the enzyme of the initial step of xylose metabolism and fermentation (Fig. 1; Table 2). Roles of enzymes in the regulation of gene expression in yeasts have been thoroughly demonstrated [46]. The specific mechanisms of Das1 and Tal2 involvements in regulation of *XYL1* remain to be elucidated, however. It cannot be excluded that peroxisomes are required for xylose to ethanol fermentations due to the location of the Das1 and Tal2 enzymes in this organelle. Indeed, in *S. stipitis*, which does not contain peroxisomal transketolase and transaldolase enzymes, peroxisomes are not required for xylose fermentation.

The ability to increase ethanol production in *O. polymorpha* from xylose after overexpression of peroxisomal transketolase and presumably peroxisomal transaldolase was shown both for the wild-type strain and for earlier constructed advanced ethanol producer from xylose [22]. Co-overexpression of Das1 and Tal2 in the advanced ethanol producer led to the accumulation of 16.0–16.5 g ethanol/L at 45 °C in xylose medium. This titer of ethanol is 30–40 × higher relative to that accumulated by the wild-type strain and one-third higher than in previous best *O. polymorpha* strain [22]. It is interesting to compare our best strain isolated herein with xylose-fermenting strain isolated in another species of thermotolerant yeasts, *Kluyveromyces marxianus* [47]. Ethanol yield and the productivity of our best *O. polymorpha* strain with overexpressed *DAS1* and *TAL2* at 45 °C are like that described for *K. marxianus* at 42 °C; however, at 45 °C, *K. marxianus* showed a drop in the mentioned parameters. Moreover, our best strain of *O. polymorpha* did not accumulate xylitol during xylose fermentation, whereas *K. marxianus* accumulated large amounts of this by-product [47]. However, the main drawback of constructed *O. polymorpha* strain is incomplete xylose consumption under fermentation condition. To address this, we plan to activate xylose uptake and manipulate the genes of glycolysis and of oxidative and non-oxidative branches of pentose phosphate pathway. Indeed, we have found recently that simultaneous overexpression of pyruvate decarboxylase and alcohol dehydrogenase in the wild-type strain of *O. polymorpha* increased ethanol accumulation from glucose, xylose, and glycerol [14]. Besides, we can expect an increase in ethanol accumulation from xylose after additional overexpression of the genes, *TKL1* and *TAL1*, coding for cytosolic transketolase and transaldolase, respectively.

## Conclusions

Xylose to ethanol fermentation in the methylotrophic thermotolerant yeast, *Ogataea polymorpha*, depends on functional peroxisomal transketolase (Das1) and transaldolase (Tal2), whereas their cytosolic counterparts (Tkl1 and Tal1) are indispensable for growth on this pentose. Defects in peroxisome biogenesis in *O. polymorpha* (but not in the non-methylotrophic yeast *S. stipitis*) strongly compromise xylose fermentation. Glucose fermentation does not depend on peroxisomes or peroxisomal and cytosolic transketolases and transaldolases. Co-overexpression of peroxisomal transketolase and transaldolase in a previously isolated, advanced ethanol from xylose producer further increased ethanol accumulation up to 16.1 g/L at 45 °C.

## Additional files

**Additional file 1.** Primers used in this study.

**Additional file 2.** Scheme of *DAS1* and *TAL2* deletion cassettes (*ZeoR* – gene conferring resistance to zeocin) and PCR verification of the correct cassette integration into genome of the wild-type strain (*das1∆* and *tal2∆* – constructed deletion strains; WT – recipient strain NCYC495 *leu 1-1*).

**Additional file 3.** Scheme of *TAL1* deletion cassette (*hphNT1* – gene conferring resistance to hygromycin) and PCR verification of the correct cassette integration into genome of the wild-type strain (*tal1∆*–constructed deletion strain; WT – recipient strain NCYC495 *leu 1-1*).

**Additional file 4.** Scheme of *TKL1* promoter replacement by *YNR1* gene promoter (*hphNT1* – gene conferring resistance to hygromycin) and PCR verification of the correct cassette integration into the genome of the wild-type strain (*pYNR1-TKL1* – constructed strains with substituted gene promoter; WT – recipient strain NCYC495 *leu 1-1*).

**Additional file 5.** Schemes of plasmids used in this study: pGLG61/DAS1, pGLG61/TAL2, pTkZr, pTaZr. (a) Expression cassettes *HpGAPpr-DAS1* and *HpGAPpr-TAL2* are shown as gray and white boxes, respectively. The geneticin resistance gene (*APH*), linked to the impaired constitutive gene promoter, encoding glyceraldehyde-3-phosphate dehydrogenase (HpGAPpr) and *O. polymorpha LEU2* gene are shown as black and light-gray boxes, respectively. The telomeric region (*TEL188*) as an autonomously replicating sequence is designated with the hatched lines. Origin of replication (ORI) and ampicillin resistance gene (bla) are shown as arrows. (b) Expression cassettes *HpGAPpr-TAL1* and *HpGAPpr-TKL1* are shown as white and gray boxes, respectively. Zeocin resistance gene (Zr), is shown as a light-gray box. (c) Expression cassettes *HpGAPpr-TAL2* and *HpGAPpr-DAS1* are shown as gray and white boxes, respectively. Nourseothricin resistance gene (*natNT2*), is shown as a black box. Restriction sites: RI, *Eco*RI; Xb, *Xba*I; PI, *Pst*I; BI, *Bam*HI; KI, *Kpn*I; BII, *Bgl*II; SmI, *Sma*I; Sc, *Sac*I; Sl, *Sal*I, Nd, *Nde*I.

**Additional file 6.** Scheme of *PEX3* deletion cassette (*HIS3*, gene involved in histidine biosynthesis, was used as selective marker) and PCR verification of the correct cassette integration into the genome of the wild-type strain (*pex3∆* – constructed deletion strain; WT – recipient strain NCYC495 *leu 1-1*).
