# Supplementary material for: Peroxisomes and peroxisomal transketolase and transaldolase enzymes are essential for xylose alcoholic fermentation by the methylotrophic thermotolerant yeast, Ogataea (Hansenula) polymorpha
Source: Biotechnol Biofuels. 2018 Jul 19;11:197. doi: 10.1186/s13068-018-1203-z (PMC6052537; doi:10.1186/s13068-018-1203-z)
Supplement: Supplementary file 1 — Additional file 1. Primers used in this study. [file 13068_2018_1203_MOESM1_ESM.docx]

| **Primers** | **Sequence 5’ – 3’** | |
| --- | --- | --- |
| **Ko770** | | CCC GAG CTC CCA AGT TAT TAG AAT TTC TGG ACG GG |
| **Ko771** | | TGC TCT AGA CTA TAA CTC GTA CAA TAT CTG GAC TGT G |
| **Ko772** | | CCA GTC TAG AAT TGA TAT AAC CCT ACT C |
| **Ko773** | | CGC GGA TCC CGG AGT GGA AAA GTG GAA ATG TCG |
| **KB5** | | TGC TCT AGA cgc gta gag aaa tgt gaa tac g |
| **KB6** | | TGC TCT AGA GGA AGA GAA TGT GAA AGG AGG |
| **Ko774** | | GTA ATA CGT AGC TCT GAT GTT GGT AGA TGC |
| **Ko775** | | CTG TTC ACC TGC CTG GGT TTC AAC TTC |
| **KB10** | | CAC TAT TCT CGC TAT TCT CTC GCT AGC TC |
| **KB10** | | gga aaa tac taa gga tac ggc tct cag tc |
| **OL207** | | TGG AAG CTT GAA TCG TGA AAC GTC GTA ATC |
| **OL208** | | GGT GAA TTC GGG AAG AAA AGA CAG AGA TGA |
| **OL209** | | TTT TCT TCC CGA ATT CAC CGG CGA CGC TCC TA |
| **OL210** | | CGG TTC TAG AGA TGC ATT CTA TTT GGT ATC GG |
| **OL211** | | TGG AAG CTT TCC CTA TTC TCT GGC GGA TTT |
| **OL212** | | TGC TGA ATT CGC TCT CTA TGT AAG TAT ACC |
| **OL213** | | GAG AGC GAA TTC AGC AAA GGC TCA GAG ATT C |
| **OL214** | | TGT GGA TCC GAT TCT GCC AAC AAA AGG TGA G |
| **Ko64** | | CCG GAA TTC TAG ACA CCA TAG CTT CAA AAT G |
| **Ko65** | | CCG GAA TTC TAG ATT CTC AAG CAA GGT TTT C |
| **Ko533** | | GGC TCT TCT GTA ACT GGT CTG |
| **Ko559** | | GAC CAC TAG AAA ACA GAT CGC TG |
| **Ko535** | | GAT CTG TCA ACC ACC TTG GAA G |
| **Ko558** | | CGT CTT GTA ACC ATA CTT CTT GTA G |
| **Ко534** | | CAA TGG AGG TCA CTG AAA GAG AAA AAA AC |
| **Ko557** | | CAC ATC CGC TCT AAC CGA AAA G |
| **Ko412** | | GTA CAA AGA TAA TAT AGA AAC AAA CCC GGG ATG AAT TCA CTT GAA TAC TTG AAA C |
| **Ko413** | | AAA CTG CAG TCA TTA TCC ACC CAG AGG CTC |
| **Ko277** | | CCG GAA TTC GGA TCC ggt att tat agc cca att atc |
| **Ko278** | | ctt tag gga ttc tca tac tca tGG TAC CTT TGT TTC TAT ATT ATC TTT GTA C |
| **Ko279** | | GTA CAA AGA TAA TAT AGA AAC AAA GGT ACC ATG AGT ATG AGA ATC CCT AAA G |
| **Ko280** | | CGC GGA TCC gat gca ttc tat ttg gta tc |
| **Ko405** | | CGC GGA TCC act cat gat ccc tgc gtc tag |
| **Ko411** | | GTT TCA AGT ATT CAA GTG AAT TCA TCC CGG GTT TGT TTC TAT ATT ATC TTT GTA C |
| **Ko412** | | gta caa aga taa tat aga aac aaa CCC GGG ATG AAT TCA CTT GAA TAC TTG AAA C |
| **Ko414** | | GGA AGA TCT TCA TTA TCC ACC CAG AGG CTC |
| **XYL1f** | | CTC ACA AGG TCA CCC CTG |
| **XYL1r** | | GAT CCT GGT TCA AAG AGC TG |
| **XYL2f** | | GCT GAG GTT GCA GAG AGA G |
| **XYL2r** | | TCC TTG CTG AAG TCG TAA GC |
| **XYL3f** | | TGA CTT GAG CGA TGC ATG C |
| **XYL3r** | | CAT TTC CAA GCT TCT CAA CG |
| **RPE1f** | | ATG ATG CCC AAG GTT GAG AC |
| **RPE1r** | | AGT TCT TTC GAC ACG TAA TCT C |
| **TAL1f** | | TGG CAG GAT GTG ACT ACT TG |
| **TAL1r** | | TTT TCA GTG GCC ATG GCA TC |
| **PDC1f** | | cac agt aca aca tga tcc ag |
| **PDC1r** | | ATC TTG GCT TGC TTG ACA AG |
| **FBP1f** | | AGA CGT GCA CAG AAC ACT G |
| **FBP1r** | | GCT TCT CTC GTG GAT CTT C |
| **PCK1f** | | ATT CTG GAT GCC ATC CAC TC |
| **PCK1r** | | GTT CTC CAT AAA CAG CTG TGC |
| **ACT1f** | | GTC ATG TCT GGT GGT ACT AC |
| **ACT1r** | | TTG GAA ATC CAC ATC TGT TGG |
| **OK74** | | CGG GGT ACC CAG CAA AGG CTC AGA GAT TC |
| **OK75** | | CTC AAA TTT AGC CTT TAG CAC GTC TAG ACA AAC TCG CCC GTA TCA G |
| **OK76** | | CTG ATA CGG GCG AGT TTG TCT AGA CGT GCT AAA GGC TAA ATT TGA G |
| **OK77** | | CGC GGA TCC GAG CTT GTA AGG GAA ACT AC |
| **OK80** | | GAT GGC AAC TGA AAA GCT ATC TGA G |
| **Ko446** | | CCG GGA TCC TCT AGA GTG ATG ACG GTG AAA ACC TCT G |
| **Ko450** | | CCG GGA TCC TCT AGA CCC AAA ACC TTC TCA AGC AAG |
| **OK81** | | GTG ACC TTA GAT CTG GGT TTA TG |
| **OK99** | | GAT CGC TAA GTA TAC TCC TCA GGA TG |
| **OK100** | | GAG CAA GGT AAC ACA GTC AGC AGA G |
| **OK170** | | CGG GGT ACC GTC TGT GCT GGA GAA GGA G |
| **OK171** | | CAT CGG GCT ACA GTT GAT TC TCT AGA GGG GAG CGC AAA GTC GGA C |
| **OK172** | | GTC CGA CTT TGC GCT CCC C TCT AGA GAA TCA ACT GTA GCC CGA TG |
| **OK173** | | GTT TGG ATA TCT TGG GAC AT GGA TCC TGT GGA GGG TAT TGT TTG TAT G |
| **OK174** | | CAT ACA AAC AAT ACC CTC CAC A GGA TCC ATG TCC CAA GAT ATC CAA AC |
| **OK175** | | CAT GCA TGC GAG CCA GCA CAG ATG GTG TC |
| **OK176** | | GAT CAT GGT AGC CGT TGG CGA C |
| **OK123** | | GGT CAG GCT CTC GCT GAA TTC |
| **OK102** | | GTA ACA CAG ACA GCT TGT AGT CAG |
| **OK72** | | GAA CTT ATT CAT CTT ACT TAA TCA TAC TTT ATC GTG GTT AGG C |
| **OK73** | | GCC TAA CCA CGA TAA AGT ATG ATT AAG TAA GAT GAA TAA GTT C |
| **A35** | | GG AAGCTT GCG GCC GC TTT GTT TCT ATA TTA TC |
| **A58** | | CGC GAGCTC cca att atc att aat aat cac |
| **K43** | | CGC GGATCC caa tta tca tta ata atc |
| **Ko52** | | TT GCGGCCGC atg tcc caa gat atc caa ac |
| **Ko53** | | CCC GAGCTC ttt tcg tcc gac ctg ttt g |
| **Ko76** | | gat aat ata gaa aca aaa tga gct cac ttg aac aac |
| **Ko77** | | GTT GTT CAA GTG AGC TCA TTT TGT TTC TAT ATT ATC |
| **Ko84** | | CGC GGATCC ttg ctg aca aac gaa act c |
