# Supplementary material for: Peroxisomes and peroxisomal transketolase and transaldolase enzymes are essential for xylose alcoholic fermentation by the methylotrophic thermotolerant yeast, Ogataea (Hansenula) polymorpha
Source: Biotechnol Biofuels. 2018 Jul 19;11:197. doi: 10.1186/s13068-018-1203-z (PMC6052537; doi:10.1186/s13068-018-1203-z)
Supplement: Supplementary file 2 — Additional file 2. Scheme of DAS1 and TAL2 deletion cassettes (ZeoR – gene conferring resistance to zeocin) and PCR verification of the correct cassette integration into genome of the wild-type strain (das1∆ and tal2∆ – constructed deletion strains; WT – recipient strain NCYC495 leu 1-1). [file 13068_2018_1203_MOESM2_ESM.pptx]

## Slide 1
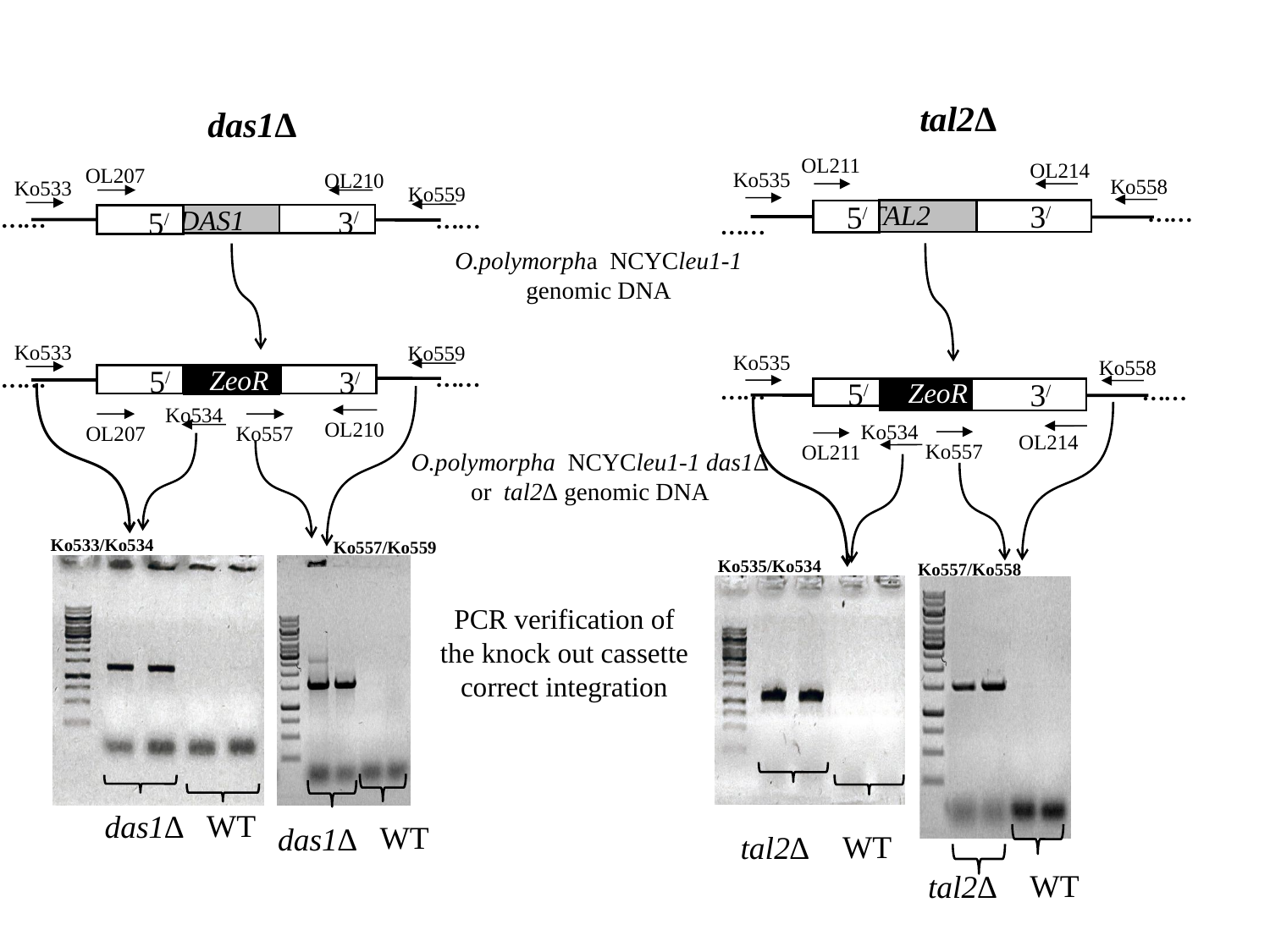

tal2∆
das1∆
OL207
OL210
Ko533
Ko559
……
……
3/
5/
 DAS1
Ko533
Ko559
……
……
5/
3/
ZeoR
Ko534
OL210
OL207
Ko557
Ko533/Ko534
Ko557/Ko559
WT
das1∆
WT
das1∆
OL211
OL214
Ko535
Ko558
……
3/
5/
 TAL2
……
Ko535
Ko558
……
……
5/
3/
ZeoR
Ko534
OL214
Ko557
OL211
Ko557/Ko558
WT
tal2∆
WT
tal2∆
O.рolymorpha NCYCleu1-1 genomic DNA
O.рolymorpha NCYCleu1-1 das1∆ or tal2∆ genomic DNA
Ko535/Ko534
PCR verification of the knock out cassette correct integration
