# Supplementary material for: Peroxisomes and peroxisomal transketolase and transaldolase enzymes are essential for xylose alcoholic fermentation by the methylotrophic thermotolerant yeast, Ogataea (Hansenula) polymorpha
Source: Biotechnol Biofuels. 2018 Jul 19;11:197. doi: 10.1186/s13068-018-1203-z (PMC6052537; doi:10.1186/s13068-018-1203-z)
Supplement: Supplementary file 3 — Additional file 3. Scheme of TAL1 deletion cassette (hphNT1 – gene conferring resistance to hygromycin) and PCR verification of the correct cassette integration into genome of the wild-type strain (tal1∆–constructed deletion strain; WT – recipient strain NCYC495 leu 1-1). [file 13068_2018_1203_MOESM3_ESM.pptx]

## Slide 1
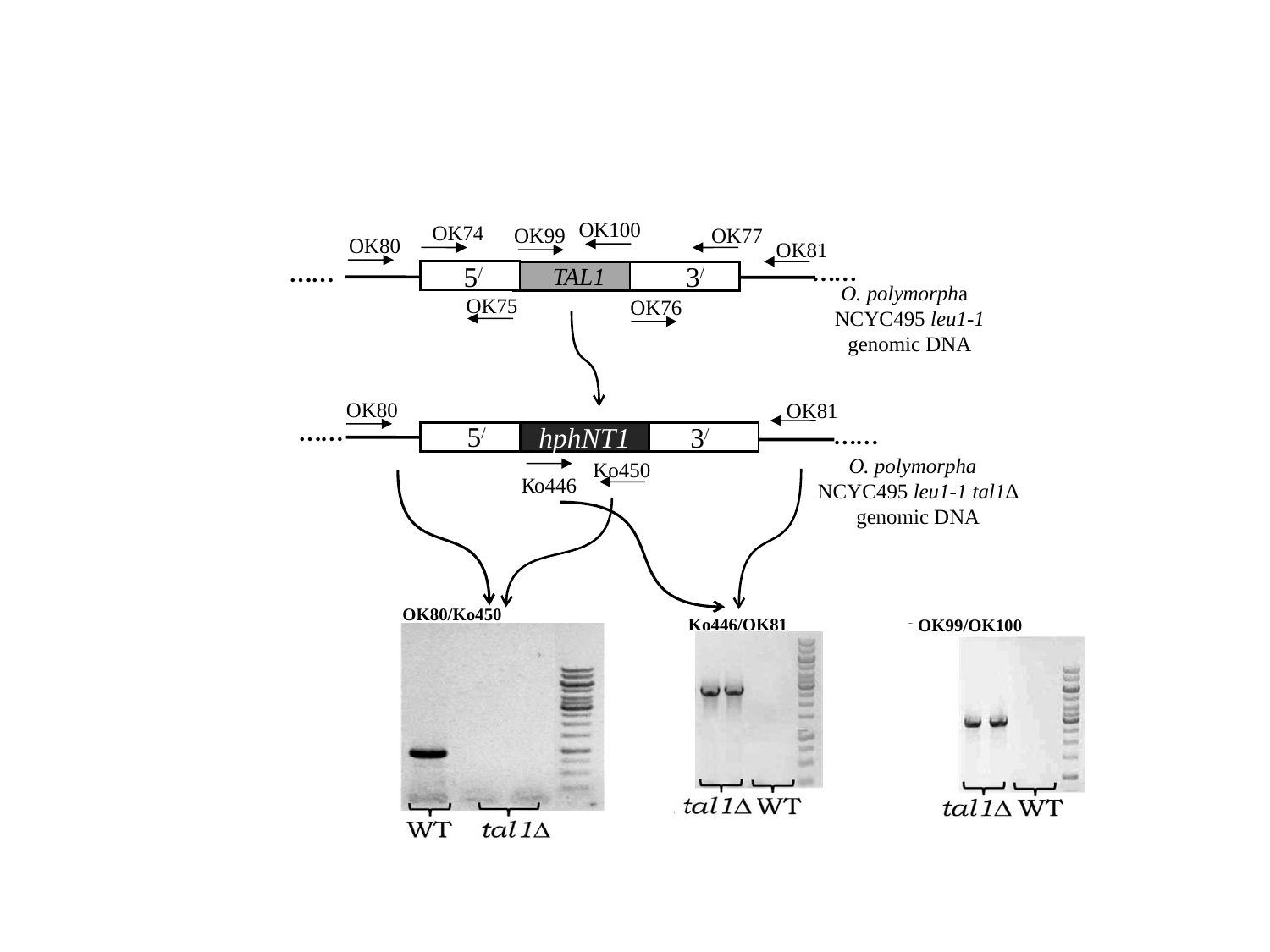

OK100
OK74
OK77
OK80
OK81
……
……
 TAL1
5/
3/
OK80
OK81
……
……
5/
3/
hphNT1
Ko450
Ко446
OK99
O. рolymorpha
NCYC495 leu1-1
genomic DNA
O. рolymorpha
NCYC495 leu1-1 tal1∆
genomic DNA
OK80/Ko450
Ko446/OK81
OK99/OK100
OK75
OK76
