# Supplementary material for: Peroxisomes and peroxisomal transketolase and transaldolase enzymes are essential for xylose alcoholic fermentation by the methylotrophic thermotolerant yeast, Ogataea (Hansenula) polymorpha
Source: Biotechnol Biofuels. 2018 Jul 19;11:197. doi: 10.1186/s13068-018-1203-z (PMC6052537; doi:10.1186/s13068-018-1203-z)
Supplement: Supplementary file 4 — Additional file 4. Scheme of TKL1 promoter replacement by YNR1 gene promoter (hphNT1 – gene conferring resistance to hygromycin) and PCR verification of the correct cassette integration into the genome of the wild-type strain (pYNR1-TKL1 – constructed strains with substituted gene promoter; WT – recipient strain NCYC495 leu 1-1). [file 13068_2018_1203_MOESM4_ESM.pptx]

## Slide 1
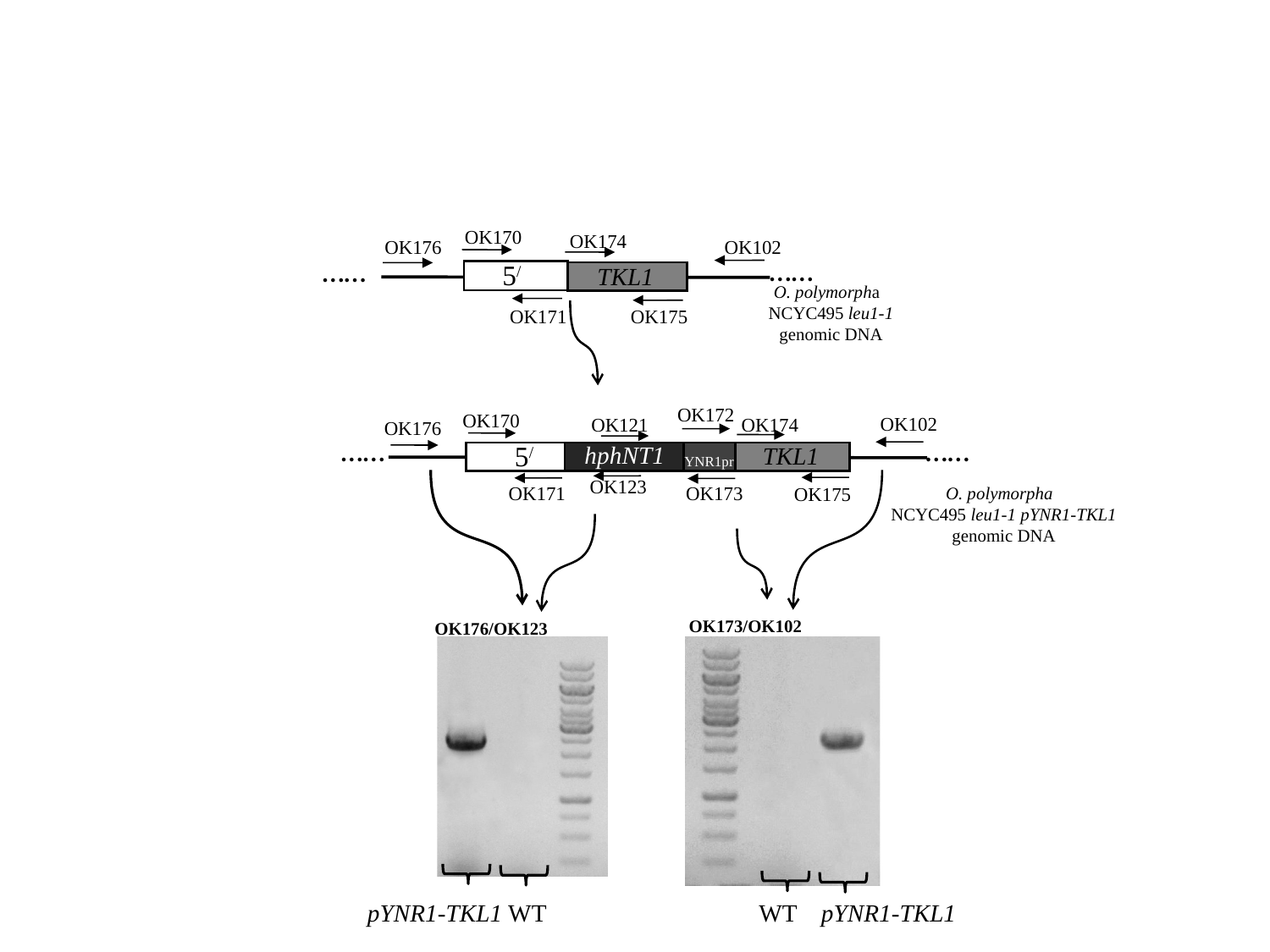

OK170
OK174
OK176
OK102
……
……
5/
 TKL1
O. рolymorpha
NCYC495 leu1-1
genomic DNA
OK171
OK175
OK172
OK170
OK102
OK121
OK174
OK176
……
……
5/
hphNT1
 TKL1
YNR1pr
OK123
OK171
OK173
OK175
O. рolymorpha
NCYC495 leu1-1 pYNR1-TKL1
genomic DNA
OK173/OK102
OK176/OK123
WT pYNR1-TKL1
pYNR1-TKL1 WT
