# Supplementary material for: Peroxisomes and peroxisomal transketolase and transaldolase enzymes are essential for xylose alcoholic fermentation by the methylotrophic thermotolerant yeast, Ogataea (Hansenula) polymorpha
Source: Biotechnol Biofuels. 2018 Jul 19;11:197. doi: 10.1186/s13068-018-1203-z (PMC6052537; doi:10.1186/s13068-018-1203-z)
Supplement: Supplementary file 5 — Additional file 5. Schemes of plasmids used in this study: pGLG61/DAS1, pGLG61/TAL2, pTkZr, pTaZr. (a) Expression cassettes HpGAPpr-DAS1 and HpGAPpr-TAL2 are shown as gray and white boxes, respectively. The geneticin resistance gene (APH), linked to the impaired constitutive gene promoter, encoding glyceraldehyde-3-phosphate dehydrogenase (HpGAPpr) and O. polymorpha LEU2 gene are shown as black and light-gray boxes, respectively. The telomeric region (TEL188) as an autonomously replicating sequence is designated with the hatched lines. Origin of replication (ORI) and ampicillin resistance gene (bla) are shown as arrows. (b) Expression cassettes HpGAPpr-TAL1 and HpGAPpr-TKL1 are shown as white and gray boxes, respectively. Zeocin resistance gene (Zr), is shown as a light-gray box. (c) Expression cassettes HpGAPpr-TAL2 and HpGAPpr-DAS1 are shown as gray and white boxes, respectively. Nourseothricin resistance gene (natNT2), is shown as a black box. Restriction sites: RI, EcoRI; Xb, XbaI; PI, PstI; BI, BamHI; KI, KpnI; BII, BglII; SmI, SmaI; Sc, SacI; Sl, SalI, Nd, NdeI. [file 13068_2018_1203_MOESM5_ESM.pptx]

## Slide 1
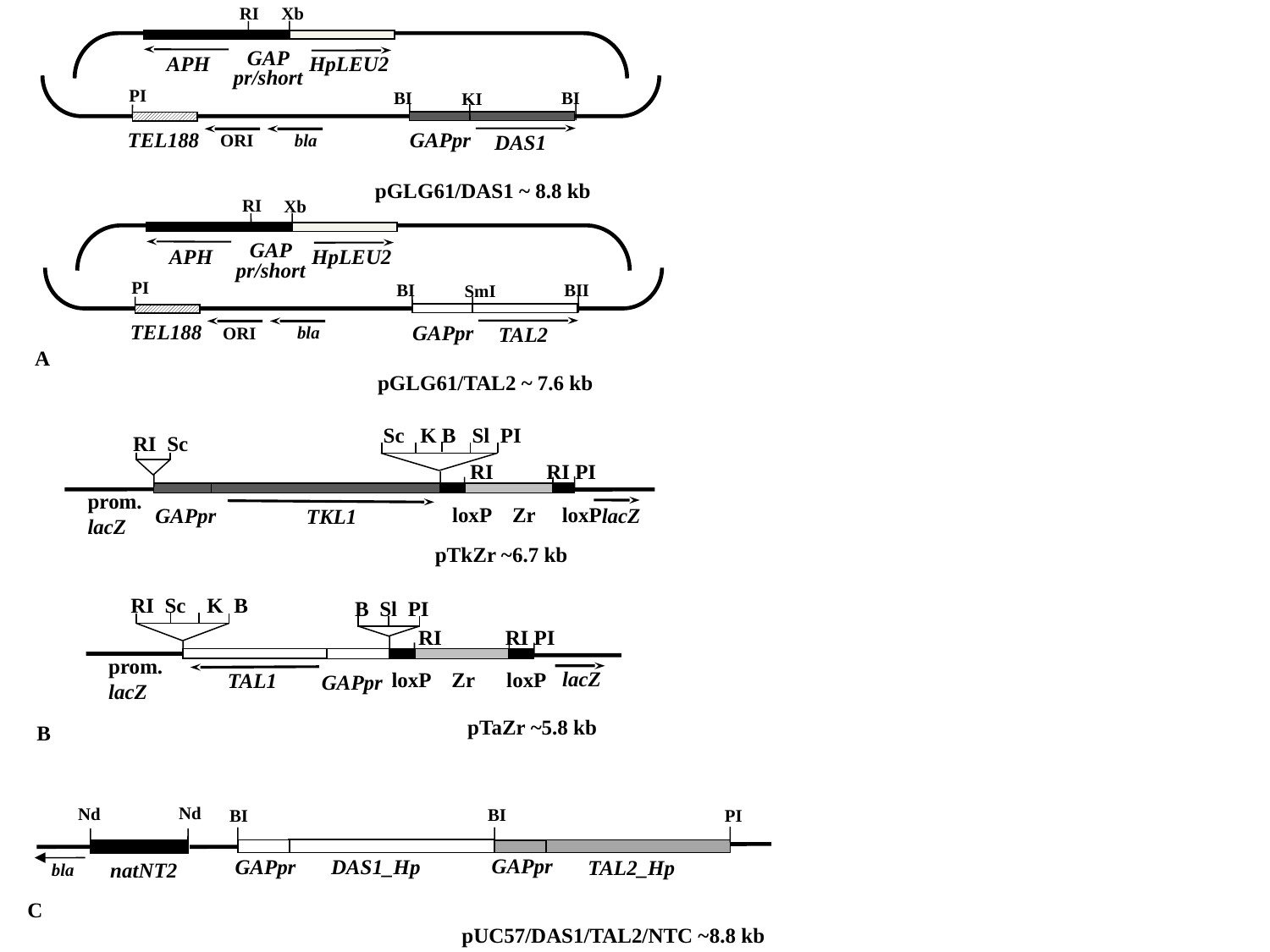

RI
Xb
GAP
pr/short
HpLEU2
APH
PI
BI
BI
KI
TEL188
bla
ORI
GAPpr
DAS1
pGLG61/DAS1 ~ 8.8 kb
RI
Xb
GAP
pr/short
HpLEU2
APH
PI
BI
BII
SmI
TEL188
bla
ORI
GAPpr
TAL2
pGLG61/TAL2 ~ 7.6 kb
A
 Sc K B Sl PI
RI Sc
RI RI PI
prom.
lacZ
lacZ
TKL1
GAPpr
pTkZr ~6.7 kb
 RI Sc K B
 B Sl PI
RI RI PI
prom.
lacZ
lacZ
loxP Zr loxP
TAL1
GAPpr
pTaZr ~5.8 kb
loxP Zr loxP
B
Nd
Nd
BI
PI
TAL2_Hp
GAPpr
BI
DAS1_Hp
GAPpr
natNT2
bla
 pUC57/DAS1/TAL2/NTC ~8.8 kb
C
