# Supplementary material for: Peroxisomes and peroxisomal transketolase and transaldolase enzymes are essential for xylose alcoholic fermentation by the methylotrophic thermotolerant yeast, Ogataea (Hansenula) polymorpha
Source: Biotechnol Biofuels. 2018 Jul 19;11:197. doi: 10.1186/s13068-018-1203-z (PMC6052537; doi:10.1186/s13068-018-1203-z)
Supplement: Supplementary file 6 — Additional file 6. Scheme of PEX3 deletion cassette (HIS3, gene involved in histidine biosynthesis, was used as selective marker) and PCR verification of the correct cassette integration into the genome of the wild-type strain (pex3∆ – constructed deletion strain; WT – recipient strain NCYC495 leu 1-1). [file 13068_2018_1203_MOESM6_ESM.pptx]

## Slide 1
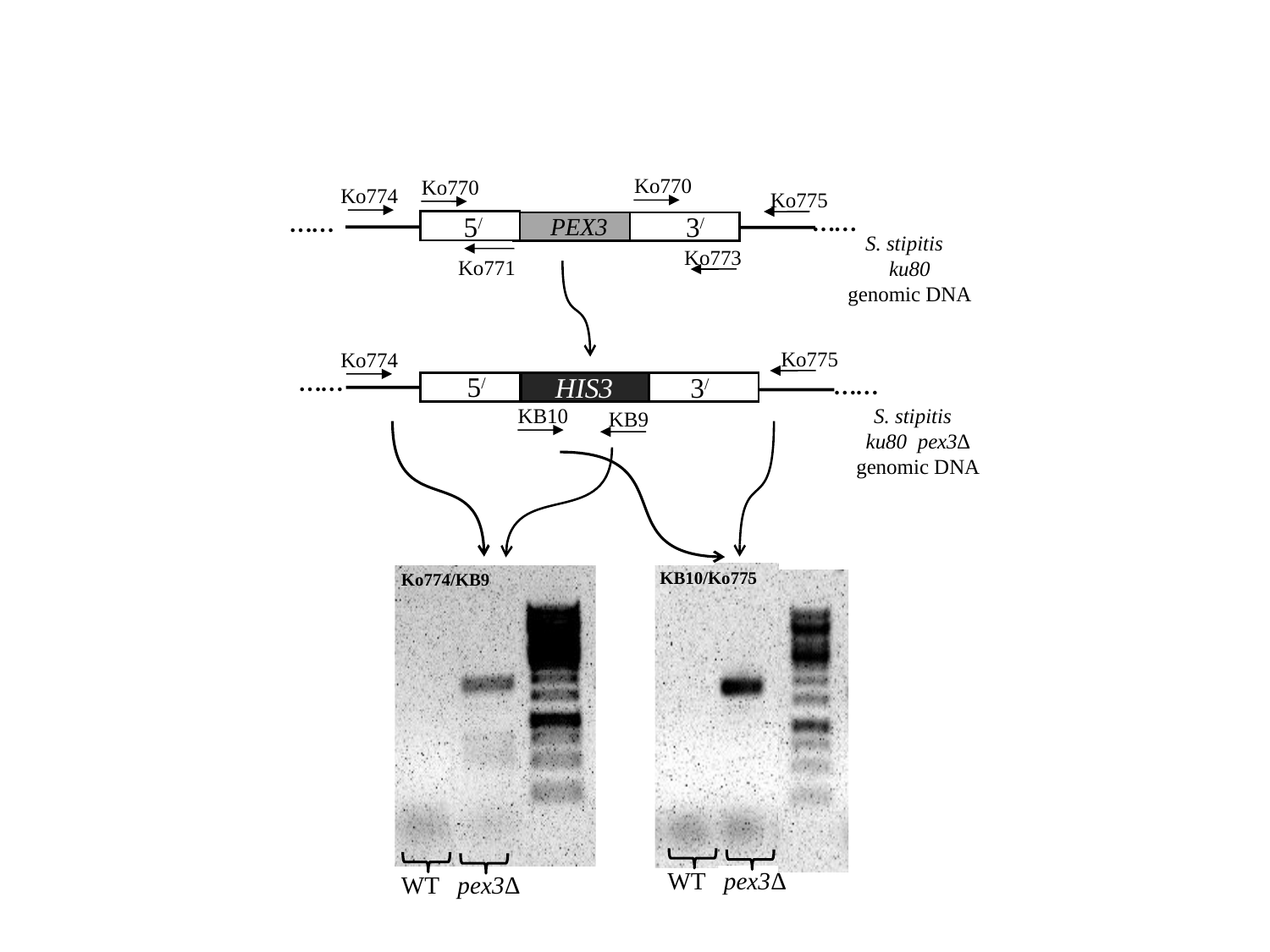

Ko770
Ko770
Ko774
Ko775
……
……
 PEX3
5/
3/
Ko775
Ko774
……
……
5/
3/
HIS3
KB10
KB9
S. stipitis
ku80
genomic DNA
Ko773
Ko771
S. stipitis
ku80 pex3∆
genomic DNA
KB10/Ko775
Ko774/KB9
WT pex3Δ
WT pex3Δ
